# SNHG12: An LncRNA as a Potential Therapeutic Target and Biomarker for Human Cancer

**DOI:** 10.3389/fonc.2019.00901

**Published:** 2019-09-18

**Authors:** Suraksha Tamang, Varnali Acharya, Deepronil Roy, Rinka Sharma, Apeksha Aryaa, Uttam Sharma, Akanksha Khandelwal, Hridayesh Prakash, Karen M. Vasquez, Aklank Jain

**Affiliations:** ^1^Department of Animal Sciences, Central University of Punjab, Bathinda, India; ^2^Department of Biochemistry and Microbial Sciences, Central University of Punjab, Bathinda, India; ^3^Department of Virology and Immunology, Amity University, Noida, India; ^4^Division of Pharmacology and Toxicology, Dell Pediatric Research Institute, College of Pharmacy, The University of Texas at Austin, Austin, TX, United States

**Keywords:** long non-coding RNA, lncRNA, SNHG12, miRNA, cancer, microRNA, small nucleolar host gene 12

## Abstract

Limitations in current diagnostic procedures warrant identification of new methodologies to improve diagnoses of cancer patients. In this context, long non-coding RNAs (lncRNAs) have emerged as stable biomarkers which are expressed abundantly in tumors. Importantly, these can be detected at all stages of tumor development, and thus may provide potential biomarkers and/or therapeutic targets. Recently, we suggested that aberrant levels of lncRNAs can be used to determine the invasive and metastatic potential of tumor cells. Further, direct correlations of lncRNAs with cancer-derived inflammation, metastasis, epithelial-to-mesenchymal transition, and other hallmarks of cancer indicate their potential as biomarkers and targets for cancer. Thus, in this review we have discussed the importance of small nucleolar RNA host gene 12 (SNHG12), a lncRNA, as a potential biomarker for a variety of cancers. A meta-analysis of a large cohort of cancer patients revealed that SNHG12 may also serve as a potential target for cancer-directed interventions due to its involvement in unfolded protein responses, which many tumor cells exploit to both evade immune-mediated attack and enhance the polarization of effector immune cells (e.g., macrophages and T cells). Thus, we propose that SNHG12 may serve as both a biomarker and a druggable therapeutic target with promising clinical potential.

## Introduction

Although excellent progress has been made in the management of cancer over the past decade, unfortunately cancer-related deaths have not significantly decreased worldwide (1). Potential reasons for this may include the invasive nature of tumor cells, the heterogeneous steps involved in cancer initiation, progression, and metastasis, including the interlinking of various signaling molecules and pathways, resulting in poor detection and treatment of cancer. In this regard, regulatory molecules such as long non-coding RNAs (lncRNAs), micro RNAs (miRNAs), piwiRNAs (piRNAs), and other non-coding RNAs (ncRNAs) comprise ~90% of the human genome. Multiple studies have demonstrated that lncRNAs are indeed functional elements involved in a wide range of physiological and pathophysiological processes, and can act as oncogenes or tumor suppressor genes (2–6).

One such lncRNA, small nucleolar host gene 12 (SNHG12), also known as LNC04080, is an lncRNA located at the p35.3 region on chromosome 1. It is ~1,867 bases long (7) and encodes four small nucleolar RNAs (SNORA66, SNORA61, SNORA16A, and SNORD99) from its spliced introns (8). Studies have implicated SNHG12 in a number of cancers, such as breast (9), gastric (10), osteosarcoma (11), and glioma (12) and other cancer types detailed in this review. The altered expression of SNHG12 has been correlated with the viability, proliferation, metastasis, and invasion of tumor cells, impacting the prognosis and survival of cancer patients.

**Figure 1:**
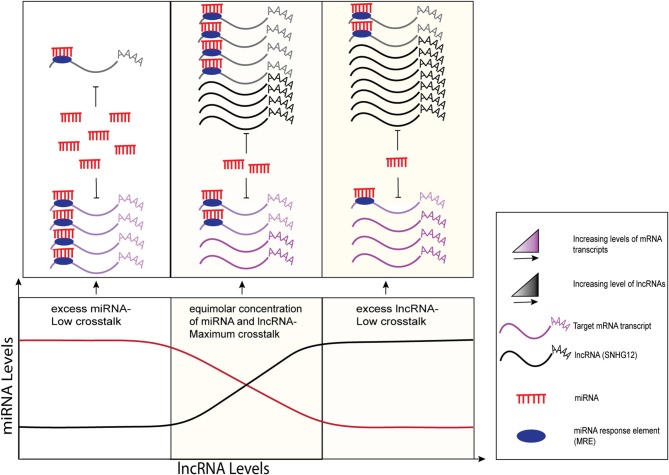
Graphical representation of sponging mechanisms of lncRNAs and miRNAs. MiRNA-mediated crosstalk depends on the relative abundance of its target transcripts. LncRNAs (black) regulate the expression levels of their target mRNA transcripts (purple) by competing for common miRNAs (red) via the MREs (filled dark blue ovals). Minimal crosstalk is observed when miRNAs are in excess (left side) or in limited numbers (right side) as compared to lncRNAs. At equimolar concentrations, maximum crosstalk is observed where lncRNAs sponge miRNAs, preventing them from binding to and repressing their target mRNAs. The figure modified from Ala et al. (17) and Tan et al. (20).

SNHG12 also acts as a competitive endogenous RNA (ceRNA) by harboring multiple miRNA binding sites, thereby regulating their downstream targets by “sponging” those miRNAs. Recent research describes the emerging role of ceRNAs in cancer etiology where various ncRNA molecules including lncRNAs, miRNAs, pseudogenes, and circular RNAs (circRNAs) share common miRNA response elements (MREs), thereby mutually regulating each other through cellular processes via complex RNA networks (13). MREs have been found in the 5'UTRs, coding sequences, and 3'UTRs of genes (14, 15). It has been reported that SNHG12 lncRNA has a higher density of MREs for target miRNAs, thereby increasing the likelihood of sharing and titrating miRNAs and preventing them from binding to other transcripts (16). According to various models, this miRNA-mediated lncRNA and mRNA crosstalk can be unidirectional, where changes in the levels of one transcript (ceRNA) can derepress the expression of the target transcript by miRNA sponging, as well as bidirectional, where both transcripts compete for the binding of miRNAs and regulate each other (17–19) as shown in Figure 1. Collectively, it has been suggested that optimal miRNA-mediated crosstalk depends on the relative abundance of participating molecules, i.e., lncRNAs and miRNAs, and maximum sponging activity is typically observed at equimolar concentrations of miRNAs and their target lncRNAs (Figure 1, middle panel). Examples of lncRNAs acting as ceRNAs for mRNA and miRNA have been reported in several publications (21, 22). For instance, highly up-regulated in liver cancer (HULC) and papillary thyroid carcinoma susceptibility candidate 3 (PTCSC3) transcripts contain miRNA-binding sites that inhibit the expression of the miRNAs and regulate downstream targets that aid in the progression of hepatocellular carcinoma (23) and thyroid cancer (24), respectively.

**Figure 2:**
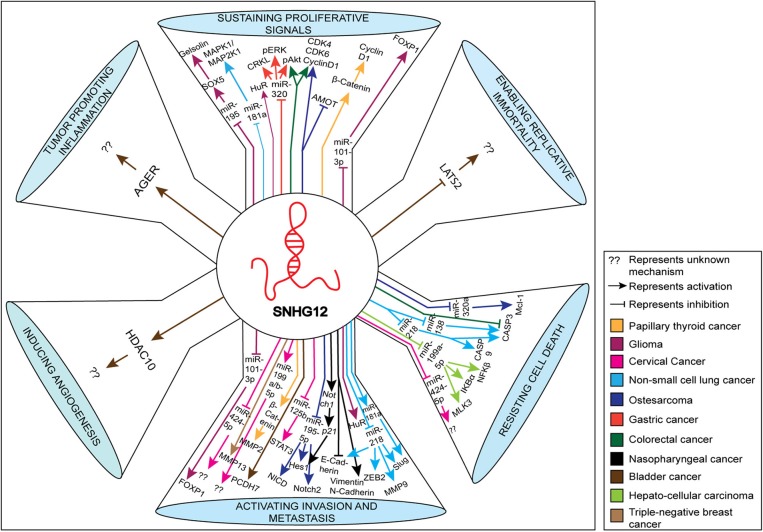
Schematic representation of various molecules and pathways targeted by SNHG12 in cancer. The various pathways shown represent hallmarks of cancer. The up-regulated and down-regulated molecules/pathways are depicted by up and down arrows, respectively.

In this review, we discuss the proposed mechanistic roles of SNHG12 in the pathogenesis of several human cancers, including the regulatory molecules of SNHG12 in modulating several hallmarks of cancer. These include interactions that: (1) *sustain proliferative signals* via inhibition of miR-195 (12), miR-181a (25), miR-320 (10), miR-101-3p (26), and up-regulation of Gelsolin (12), Mitogen-Activated Protein Kinase 1/Mitogen-Activated Protein Kinase Kinase1 (MAPK1/MAP2K1) (25), phosphorylated Extracellular Signal-Related Kinase (pERK) (10), phosphorylated Protein Kinase B (pAkt) (10), Cyclin D1 (11, 27), and Forkhead Box P1 (FOXp1) (26); (2) *enable replicative immortality* via inhibition of Large Tumor Supressor Kinase 2 (LATS2) (28); (3) *resist cell death* via inhibition of miR-320a (29), Caspase 3 (30), miR-138 (31), miR-218 (32), miR-199a-5p (8), miR-424-5p (33), and up-regulation of MCL1 Apoptosis Regulator, BCL2 family member (Mcl-1) (29), Caspase 9 (32), Mixed Lineage Protein Kinase 3 (MLK3) (8), NF-KB inhibitor alpha (IKBα) (8), NFKB inhibitor beta (IKBβ) (8); (4) *activate invasion and metastasis* via inhibition of miR-101-3p (26), miR-424-5p (33), miR-125b (34), miR-195-5p (11), E-Cadherin (35), miR-218 (32), and upregulation of Forkhead Box P1 (FOXP1) (26), Matrix Metallopeptidase 3 (MMP13) (9), Matrix Metallopeptidase 2 (MMP2) (27), Signal Transducer and Activator of Transcription (STAT3) (34), Notch2 (11), Vimentin (35), N-Cadherin (35), Zinc Finger E-Box Binding Homeobox (ZEB2) (32), Matrix Metallopeptidase 9 (MMP9) (32), and Slug (32); (5) *induce angiogenesis* via targeting Histone Deacetylase 10 (HDAC10) (28); and (6) *promote inflammation* via targeting the Advanced Glycosylation End-Product Specific Receptor (AGER) (28). The diagrammatic representation of the impact of SNHG12 in these mechanisms and hallmarks of cancer are shown in Figure 2. We have highlighted the target molecules of SNHG12, which may serve as potential diagnostic and prognostic biomarkers, and/or as targets for novel strategies for cancer therapy.

## Association of SNHG12 in Various Cancer Types

### Gastric Cancer (GC)

SNHG12 expression was evaluated by two different research groups in over 100 GC patients combined, and they found an ~1.6-fold up-regulation of SNHG12 in GC tissues compared to adjacent normal tissues (10, 36). In both the studies, SNHG12 expression was positively correlated with tumor size, stage, lymph node metastasis, and distant metastasis; whereas, no correlation was found with other clinicopathological characteristics such as patient age and gender. Moreover, its high expression was negatively correlated with patient survival time and disease-free survival (10, 36). Similarly, the up-regulation of SNHG12 (up to ~15-fold) was observed in the GC cell lines SGC-7901, AGS, and BGC-823, compared to normal gastric epithelial (GES-1) cells, as outlined in Table 1. In addition, siRNA knockdown of SNHG12 in GC cells led to reduced proliferation and colony formation when assessed by CCK-8 staining and colony formation assays. Subsequently, both Zhang et al. (10) and Yang et al. (36) identified miR-199a/b-5p and miR-320 as potential target molecules of SNGH12 by using microRNAcode and LNCipedia database prediction analyses (Figure 2) and through dual-luciferase reporter assays they confirmed miR-199a/b-5p and miR-320 as its direct downstream targets (10, 36). Moreover, knockdown of SNHG12 in GC cell lines significantly up-regulated the levels of both the miRNAs showing a negative correlation between the two and reversing the effect of SNHG12 in cancer progression. Knockdown of SNHG12 also led to down-regulation of CRK Like Proto-Oncogene, Adaptor Protein (CRKL), p-AKT, and p-ERK proteins. Consistent with this, *CRKL* was previously reported to be a target of miR-320 in GC cells (52) (Figure 2). Importantly, miR-320-mediated *CRKL* inhibition was found to enhance cell proliferation, invasion, and metastasis of GC cells via up-regulation of the ERK and AKT pathways (52). It was suggested that SNHG12 up-regulated *CRKL* expression, thereby enhancing AKT and ERK signaling, thus promoting GC progression via sponging of miR-320 (10) (Figure 2). Thus, these studies implicated SNHG12 in GC by sponging miR-320 and miR-199a/b-5p, thereby regulating oncogenic pathways.

**Table 1:** Targets/pathways of SNHG12 in different tissues/cell lines in various cancers.

**Cancer**	**Tissues/cell lines studied**	**Approx. mean fold up-regulation compared to control samples**	**Genes/proteins/pathways affected**	**Validation methods**	**Useful for prognosis, diagnosis, and therapeutics**	**References**
Gastric cancer	Tissues, SGC-7901, AGS	1.5-fold 2.5-fold 2.7-fold	**↓**miR-320, **↑**CRKL, **↑**p-Akt, **↑**p-Erk	qRT-PCR, immunoblot	Yes	(10, 36)
	Tissues, SGC-7901, BGC-823	1.6-fold 13-fold 15-fold	**↓** miR-199a/b-5p	qRT-PCR		
Non-small cell lung cancer	H1299, A549	2.7-fold 2.2-fold	**↓**miR-138	qRT-PCR	Yes	(25, 31, 32, 37–39)
	Tissues, H1299, A549, PC9, A549/DDP, A549/PTX, PC9/AB2	2.3-fold 2.3-fold 3-fold 2.5-fold 5-fold 4.8-fold 5.5-fold	**↓**miR-181a, **↑**MAPK1, **↑**MAP2K1, **↑**Slug, **↑**MAPK/Slug pathway	qRT-PCR, immunoblot		
	Tissues	27-fold		qRT-PCR		
	Tissues	1.25-fold	**↓**miR-218, **↑**MMP9, **↑**Vimentin, **↑**Slug, **↑**ZEB2, **↓**E-Cadherin	qRT-PCR, immunoblot		
Triple-negative breast cancer	Tissues, MDA-MB-231 BT-549	2.2-fold Not determined	**↑**MMP13	qRT-PCR	Yes	(9)
Hepato-cellular carcinoma	Tissues, SK-Hep1 HCCLM9	3.7-fold Not determined	**↓**miR-199a/b-5p, **↑**MLK3, **↑** IκB, **↑**NF-κB	qRT-PCR, ChIRP assay, immunoblot	Yes	(8, 40)
Colorectal cancer	Tissues, SW480, HT29	2.5-fold 2-fold 4.7-fold	**↑**Akt, **↑**PI3K/AKT signaling pathway, **↑**CDK4, **↑** CDK6, **↑**CCND1, **↓**Caspase-3	qRT-PCR, immunoblot	Yes	(30)
Bladder cancer	Tissues, UMUC3, SW780	1.1-fold Not determined	↑HDAC10, ↑AGER, ↓PCDH7, ↓LATS2	qRT-PCR, microarray	Yes	(28)
Papillary thyroid cancer	Tissues, BCPAP, TPC1, K1	3.8-fold 6-fold 5-fold 3-fold	↑Wnt/β-catenin signaling pathway, **↑**β-catenin, **↑**MMP-2, **↑**cyclin D1	qRT-PCR, immunoblot	Yes	(27)
Glioma	Tissues, U251, U87	3.3-fold 5.3-fold 5.2-fold	↓miR-101-3p, **↑**FOXP1	qRT-PCR, immunoblot	s	(12, 26, 41)
	Tissues, U251, U87	2.2-fold 4.2-fold 3.8-fold	**↑**HuR	qRT-PCR, immunoblot		
	Tissues, U251, U87	5.5-fold 13-fold 11-fold	**↑**TDP4, **↓**miR-195, **↑**SOX5, **↑**Gelsolin	qRT-PCR, immunoblot		
Cervical cancer	Tissues, CaSki, SiHa	1.8-fold 6.3-fold 5.3-fold	**↓**miR-424-5p	qRT-PCR, immunoblot		(33, 34, 42–44)
	HeLa, SiHa	3.5-fold 3.3-fold	**↓**miR-125b, **↑**STAT3	qRT-PCR		
Nasopharyngeal cancer	Tissues, CNE1, SUNE1	1.5-fold 10-fold 7.7-fold	**↑**Notch pathway	qRT-PCR	Yes	(35)
Osteosarcoma	Tissues, MG-63	3.4-fold 3.3-fold	**↑**Angiomotin	qRT-PCR	Yes	(11, 29, 45, 46)
	Tissues, 143B, U2OS	2.9-fold 6.2-fold 5.6-fold	**↓**miR-195-5p, **↑**CDK4, **↑**CDK6, **↑**CCND1, **↑**Notch, **↑**Notch signaling	qRT-PCR		
	DXR resistant tissues, MG-63, U2OS, MG-63/DXR	2.5-fold 3.9-fold 3.8-fold 5.4-fold	**↓**miR-320a, **↑**MCL1	qRT-PCR		
Prostate cancer	Tissues LNCaP PC-3 C4-2	1.8-fold 22-fold 14-fold 11-fold	**↓**miR-133b, **↑**P53 signaling pathway	qRT-PCR	Yes	(47, 48)
	Tissues PC-3 DU145	1.8-fold 1.7-fold 2.3-fold	**↓**miR-195, **↑**β-catenin, **↑**Cyclin D1, **↑** c-Myc, **↑**Wnt/ β-catenin signaling	qRT-PCR		
Renal cell carcinoma	Tissues Caki-1 ACHN	1.75-fold 5.6-fold 5.8-fold	**↓**miR-199a-5p, **↓**PARP, **↓**Caspase-3, **↑**HIF1α	qRT-PCR	Yes	(49)
Ovarian Cancer	Tissues A2780 HO8910	1.7-fold 1.5-fold 3-fold	**↓**miR-129, **↑**SOX4	qRT-PCR	Yes	(50)
Natural killer/T-cell lymphoma	Tissues SNK-6 YTS	1.29-fold 4.6-fold 3-fold	**↑**c-Myc, **↑**P-gp	qRT-PCR	Yes	(51)

In another study, low plasma levels of SNHG12 were reported in 10 pre-operative GC patients compared to their post-operative samples and 10 healthy controls (53), which is in contrast to its expression in cancer tissue samples. Thus, additional studies are needed to understand the mechanistic roles of SNHG12 in GC to better define its potential as a candidate biomarker to facilitate early diagnosis of GC, and/or as a potential novel therapeutic target for the treatment of GC to overcome its high mortality rates (1).

### Non-small Cell Lung Cancer (NSCLC)

Several studies have documented significantly up-regulated expression of SNHG12 in NSCLC tissue samples as well as NSCLC cell lines compared to normal control samples. Zhu et al. (54) observed an ~27-fold higher expression of SNHG12 in 10 lung adenocarcinoma (LAD) tissues compared to adjacent non-cancerous tissues through qRT-PCR experiments. Similarly, two additional studies observed an ~2.3-fold and ~1.3-fold up-regulation of SNHG12 in 22 and 40 NSCLC tumor samples compared to adjacent normal tissues, respectively (25, 32). Importantly, higher expression of SNHG12 was found to be negatively associated with the overall survival rate of NSCLC patients (32). Additionally, NSCLC cell lines (PC9, H1299, and A549) were examined for SNHG12 expression, where an ~2.5-fold up-regulation in PC9 cells, up to ~2.7-fold up-regulation in H1299 cells (25, 31) and as much as an ~3-fold up-regulation in A549 cells (25, 31) was observed compared to normal human bronchial epithelial (16-HBE) cells (Table 1).

Studies have shown that SNHG12, like other lncRNAs, acts as an endogenous sponge that competes for several miRNAs including hsa-miR-181b-5p, hsa-miR-181a-5p, hsa-miR-16-5p, hsa-miR-15a-5p, hsa-miR-195-5p, hsa-miR-497-5p, hsa-miR-181c-5p, hsa-miR-181d-5p, hsa-miR-15b-5p, hsa-miR-138, and hsa-miR-218 in NSCLC (31). It was found that alterations in miR-181a, a tumor suppressor miRNA (32, 37, 55), expression levels affected its downstream targets, such as MAPK and Slug (Figure 2), facilitating the development of NSCLC (38, 39, 56, 57). Likewise, over-expression of miR-138 suppressed cell proliferation and colony formation by increasing the apoptotic rate of NSCLC cells via up-regulated Caspase-3 activity, which suppressed NSCLC progression (37). Furthermore, altered expression of miR-218 was found to be associated with increased tumorigenesis by increasing the epithelial-to-mesenchymal transition in NSCLC by targeting Matrix Metallopeptidase 9 (MMP-9), Vimentin, Slug, Zinc Finger E-Box Binding Homeobox 2 (ZEB2), and E-cadherin proteins (Figure 2) (32). In addition, miR-218 up-regulation positively correlated with the expression levels of cleaved Caspase-3 and Caspase-9, thereby suppressing cell viability and cancer progression by triggering apoptosis. Indeed, dual-luciferase reporter assays confirmed the transcriptional regulation of SNHG12 with hsa-miR-181a, hsa-miR-138, and hsa-miR-218 (25, 31, 32). At the cellular level, knockdown of SNHG12 resulted in a significant decrease in cell proliferation, colony formation, and invasive potential of cells, but increased apoptosis by up-regulating the expression of the target miRNAs. Overall, these data revealed that SNHG12 negatively regulated the expression of its target miRNAs in NSCLC (32).

These composite results support the conclusion that SNHG12 is up-regulated in NSCLC where it plays a direct role in the modulation of cancer progression through down-regulating the expression of tumor-suppressor miRNAs, such as miR-138 and miR-181a, and it is also involved in NSCLC drug resistance. Thus, SNHG12 may serve as a potential diagnostic biomarker, and therapeutic target in the treatment of NSCLC, including patients that have developed resistance to therapy.

### Triple Negative Breast Cancer (TNBC)

Wang et al. (9) have identified numerous differentially expressed lncRNAs through RNA sequencing in TNBC compared to non-tumorous breast tissues, and found an increased expression (~2.2-fold) of SNHG12 in TNBC (Table 1). Further, SNHG12 up-regulation was positively correlated with advanced tumor stage and size, while it negatively correlated with patient survival, thus demonstrating its clinical significance. Mechanistic investigation revealed that SNHG12 was a direct transcriptional target of c-MYC. Using the Multiple EM for Motif Elicitation tool for discovering motifs (MEME), the authors identified two putative c-MYC-binding motifs at 1,564 bp and 780 bp upstream of the SNHG12 transcription start site. This was confirmed when transfection of a *c-MYC*-containing plasmid increased SNHG12 levels in MDA-MB-231 and BT-549 TNBC cell lines, enhancing their viability and colony-forming capacity, increasing cell proliferation, and inhibiting apoptosis (9). Over-expression of SNHG12 also significantly increased the expression of *MMP13* (Table 1; Figure 2), while SNHG12 knockdown constrained its expression.

In conclusion, SNHG12 was up-regulated in TNBC and promoted proliferation, while inhibiting apoptosis. These results implicate SNHG12 in TNBC, suggesting that it may provide a target for the treatment and diagnosis of TNBC.

### Hepatocellular Carcinoma (HCC)

Lan et al. (8) measured SNHG12 levels in 48 human HCCs and their adjacent normal tissues, where 24 HCCs showed significant up-regulation (by ~3.7-fold) in cancerous tissues (Table 1). SNHG12 up-regulation was positively correlated with tumor size, vascular invasion, and tumor node metastasis (TNM) stage, while it was negatively correlated with patient survival, demonstrating its clinical significance. However, while SNHG12 knockdown by siRNA reduced cell viability, colony formation, and inhibited cell cycle progression, its depletion increased apoptosis of SK-Hep1 and HCCLM9 cells lines *in vitro* (8).

The suspected crosstalk between SNHG12 and miR-199a/b-5p was analyzed by miRcode software (http://www.mircode.org/), and was confirmed by a decreased luciferase activity of a pMir-reporter-SNHG12-WT when transfected with a miR-199a/b-5p mimic in HCC cell lines. Furthermore, loss-of-function assays revealed that there was an inverse correlation between the expression of miR-199a-5p and SNHG12 (Figure 2) (40, 58). When miRNAs exert silencing functions on target protein-coding genes, RISC formation can occur, allowing binding to the target via Ago2. To confirm the speculation that Ago2 could directly bind to both SNHG12 and miR-199a/b-5p, RIP and qRT-PCR analyses were conducted. The results demonstrated that SNHG12 and miR-199a/b-5p were enriched in the Ago2-containing pellet (8). Results of chromatin isolation by RNA purification assays supported these findings by demonstrating that SNHG12 co-immunoprecipitated with miR-199a/b-5p, confirming their interaction (8). The decrease in miR-199a/b-5p expression was found to be associated with increased tumorigenesis, at least in part by regulation of the mitogen-activated protein kinase 3 (MLK3)/IkB-α/NF-kB pathway (Figure 2) (59). Indeed, immunoblot analysis indicate that elevated expression of miR-199a-5p and knockdown of SNHG12 suppressed the expression of MLK3 and phosphorylation of IkB-α and NF-kB in SK-Hep1 cells (Figure 2). In contrast, the phosphorylation of ERK1/2, a key regulator of MAPK signaling pathways, was not affected (8). These results suggested that SNGH12 induced tumorigenesis in HCC by regulating the MLK3/IkB-α/NF-kB pathway (Figure 2).

### Colorectal Cancer (CRC)

Wang et al. (30) measured SNHG12 levels in 60 human CRCs and their adjacent normal tissues, which showed significant up-regulation (by ~2.5-fold) in the cancerous tissues over the normal tissues (Table 1) (60). SNHG12 up-regulation was positively correlated with advanced tumor stage and size, while it negatively correlated with patient survival (60). Similarly, levels of SNHG12 were significantly up-regulated (~4.7-fold) in the HT29 CRC cell line compared to the normal human colonic epithelial cell line (HcoEpiC) (60). Consistent with these observations, over-expression of SNHG12 in SW480 and HT29 cell lines increased cell viability, colony-forming capacity, cell proliferation, and inhibited apoptosis (60). In addition, immunoblot analysis confirmed that SNHG12 increased protein levels of CDK4, CDK6, Cyclin D1 (CCND1), and decreased Caspase-3 levels (Figure 2). The authors also reported that the over-expression of SNHG12 significantly up-regulated (~3-fold) the expression of pAKT (Figure 2), while SNHG12 depletion constrained its expression (60).

In conclusion, these results suggested that SNHG12 promoted cell growth and inhibited cell apoptosis in CRC cells. Thus, it may serve as a potential diagnostic biomarker and therapeutic target and for the detection and treatment of CRC.

### Bladder Cancer

Jiang et al. (28) evaluated data on expression of lncRNAs in bladder cancer specimens from The Cancer Genome Atlas (TCGA) and GSE89006 datasets and found the expression of SNHG12 to be up-regulated as compared to the normal adjacent tissues by ~1.1 and ~1.2-fold, respectively (Table 1). The higher expression of SNHG12 was found to be associated with relatively shorter recurrence-free survival time (RFS). Moreover, RNA sequencing data from the Cancer Cell Line Encyclopedia (CCLE) revealed higher expression of SNHG12 in the bladder cancer cell lines SW780 and UMUC3, compared to normal control cell lines. Depletion of SNHG12 in SW780 and UMUC3 cell lines by siRNA resulted in impaired cellular proliferation, colony formation, and invasion potential of bladder cancer cell lines (28). Furthermore, it was observed that siRNA-mediated knockdown of SNGH12 in SW780 bladder cancer cells led to a ~9% increase in the apoptotic rate of the SW780 cells compared to control cells. SNGH12 knockdown also impaired the invasive capacity of SW780 cells compared to control cells (28). These data suggested a role for SNGH12 in bladder cancer progression and invasion. The TCGA and Gene Ontology (GO) analyses revealed that SNHG12 expression was positively associated with protein-coding genes such as *Histone Deacetylase 10 (HDAC10) and Advanced Glycosylation End-product Specific Receptor (AGER), and negatively associated with other protein-coding genes such as Protocadherin 7 (PCDH7) and Large Tumor-Suppressor Kinase 2 (LATS2)* (Figure 2), which are involved in biological pathways that assist in regulating cancer cell growth and proliferation.

These results provided evidence that SNHG12 up-regulation, which associated positively with the expression of certain oncogenes and inversely with tumor-suppressor genes, plays an essential role in the growth and proliferation of bladder cancer. The increased expression of SNHG12 also correlated with shorter survival time of patients, suggesting that it may be utilized as a potential biomarker for the improved prognosis, and as a target for the treatment of bladder cancer.

### Papillary Thyroid Cancer (PTC)

Ding et al. (27) examined 42 PTC tissues and found SNHG12 to be up-regulated by ~3.8-fold in the PTC tissues compared to normal adjacent tissue samples. Similarly, in the PTC cell lines K1, TPC-1, and BCPAP, the expression of SNHG12 was found to be up-regulated by ~3, ~5, and ~6-fold over controls, respectively (Table 1) (27). Decreased SNHG12 expression by SNHG12 interference sequences in the cancer cell lines, TPC-1, and BCPAP, resulted in decreased proliferative capacity, an increased apoptotic rate (~28% increase in K1 and >28% in TPC-1), and impaired metastatic and migration capacities, implicating SNHG12 in tumor cell proliferation. Further, the results of immunoblotting revealed that SNHG12 promoted PTC tissue proliferation and metastasis, in part, through Wnt/β-catenin signaling by up-regulating β-catenin and the downstream genes, *Matrix Metalloproteinase 2 (MMP-2)* and *CCND1* (Figure 2). Similar results were found in *in vivo* experiments performed in BALB/c male nude mice, which demonstrated that knockdown of SNHG12 in the experimental group led to decreased numbers of metastatic tumors in the lungs of the mice carrying BCPAP cell xenografts compared with the control group (27).

Based on the findings mentioned above, it can be inferred that SNHG12 plays an important role in the proliferation and metastasis of papillary thyroid cancer tissues and cells by regulating the Wnt/β-catenin signaling pathway. Thus, it adds to the list of tumor types in which SNHG12 may serve as an effective tool in the diagnosis and treatment of cancer.

### Glioma

The expression of SNHG12 was examined by three different research groups in 33, 39, and 79 glioma tissue samples compared to adjacent normal tissues and were found to be up-regulated by ~3.3, ~5.5, and ~2.2-fold, respectively (Table 1) (12, 26, 41). The over-expression of SNHG12 was found to be associated with low overall survival and clinicopathological characteristics of patients such as age, WHO grade, and Karnofsky Performance Score (KPS) (26). Moreover, over-expression of SNHG12 was found to be associated with genotypes having mutations in the *telomerase reverse transcriptase (TERT)* gene and the *isocitrate dehydrogenase* ½ *(IDH1/2)* genes, along with 1p/19q co-deletion, and methylation of O-6-Methylguanine-DNA Methyltransferase (MGMT) (42). Additionally, RNA-binding proteins *Tar DNA Binding Protein (TDP-43)* and *Hu-Antigen R (HuR)* were reported to play oncogenic roles in the progression of glioma by binding to and stabilizing SNHG12 (12, 41). In three independent studies SNHG12 was found to be over-expressed by ~5.2, ~3.8, and ~11-fold in the U87 glioma cell line, and ~5.3, ~4.2, and ~13-fold in the U251 glioma cell line (Table 1) (12, 26, 41). Knockdown of SNHG12 in the U87 and U251 glioma cells resulted in decreased cell numbers, impaired proliferation, migration, and invasion, but increased the apoptotic rate in these cells compared to control human astrocytes (12, 26, 41). Similar results were obtained from *in vivo* experiments conducted in athymic BALB/c nude mice, where in one study, lentivirus-mediated transfection with sh-RNA against SNHG12 decreased tumor weight and volume of human U87 glioma cell xenografts in mice (26).

As previously mentioned, SNHG12 was found to act as ceRNA capable of sponging several miRNAs that serve as tumor suppressors in various cancers (11, 33, 61). MiR-101-3p and miR-195 were found to be putative targets of SNHG12, which inhibited the progression of glioma by down-regulating their target genes, *Forkhead Box Protein P1 (FOXP1), and Sry-Box 5 (SOX5)* (12, 26). A negative correlation was found between SNHG12 and its target miRNAs (miR-101-3p and miR-195) by qRT-PCR and immunoblot analyses, which demonstrated upregulation of the downstream targets (12, 26). Further, luciferase-reporter and RIP assays confirmed the direct interaction of SNHG12 with the above-mentioned miRNA targets, as well as their involvement in the formation of RISC complexes. *FOXP1*, a transcriptional factor in the FOXP subfamily of the FOX family, impacts the cell cycle, cell proliferation, differentiation, apoptosis, and has played an oncogenic role in various B-cell lymphomas (26). Similarly, SOX5, a transcription factor of the group D of sex-determining region (Sry)-related transcription factors (*SOXD*) protein family, acts as a transcriptional regulator and has been implicated in tumor progression. In addition, SOX5 was found to regulate the malignant progression of glioma cells via direct binding to the promoter of its downstream target gene *Gelsolin*, and the promoter of SNHG12. Consistent with these reports, knockdown of *FOXP* and *SOX5* hindered cell proliferation, invasion, and migration potential in U87 and U251 cell lines, suggesting their oncogenic function in glioma cells.

Taken together, these findings suggest that SNHG12 acts as a key player in the progression of glioma by its involvement in the miR-101-3p/*FOXP1* axis, and the miR-195/*SOX5* axis, which are stabilized by the binding of the RNA-binding proteins, HuR and TDP43, respectively. Thus, SNHG12 may serve as a potential biomarker/target for glioma.

### Cervical Cancer

Dong et al. (34) measured the expression of SNHG12 in 76 paired cervical cancer tissues and found that it was up-regulated (by ~1.8-fold) compared to adjacent normal tissues. Increased levels of SNHG12 were found to be associated with clinicopathological manifestations such as lymph node metastasis, and International Federation of Gynecology and Obstetrics (FIGO) stage, where high-grade tumors (stage II) showed increased expression compared to low-grade tumors (stage I). High SNHG12 levels were also found to be negatively correlated with the overall survival of patients (34). Similarly, five different cervical cell lines showed increased levels of SNHG12 in two separate studies compared to the normal control cell lines viz. ~6-fold in CaSKi cells and ~5-fold in SiHa cells as compared to the control NC104 cells (34), and ~3.5-fold in HeLa cells and ~3.3-fold in SiHa cells as compared to the immortalized cervical epithelial cell line, ICE (33) (Table 1).

In addition to these observations, it was found that SNHG12 promoted cervical cell motility and aided in their proliferation and migration by regulating downstream targets via sponging of miR-424-5p and miR-125b, as outlined in Figure 2 (33, 34). According to previous reports, miR-424-5p functions as a tumor suppressor by enhancing apoptosis of cancer cells and blocking the G1/S transition (43). Similarly, miR-125b can also inhibit tumor growth by suppressing the expression of the anti-apoptotic gene, *BCL-2*, consequently enhancing apoptosis (44). These effects were apparent when shRNA-mediated knockdown of SNHG12 impeded cell metastasis in HeLa, SiHa, and CaSki cervical cell lines, corresponding to up-regulated levels of miR-125b and miR-424-5p (33). In an *in vivo* study, knockdown of SNHG12 resulted in smaller tumor volumes and reduced tumor weights in CaSki cervical cancer cell-derived human xenografts in nude mice compared to the control mice (34). Further analyses implied that the *signal transducer and activator of transcription* (*STAT3*) gene acted as a direct downstream target of miR-125b (Figure 2) (42); *STAT3* is known to function both as a transcription factor and an oncogene (62). It was found that phosphorylated STAT3 elevated the levels of the anti-apoptotic genes *BCL-2 like 1 (BCL-XL), Survivin, and Myeloid Cell Leukemia Sequence 1 (MCL-1)* in endometrial and cervical cancer tissues relative to normal tissue samples, aiding in their progression (63). Consistent with these reports, qRT-PCR results and immunoblot analyses revealed that miR-125b mimicked repressed *STAT3* expression in cervical cancer cell lines (HeLa and SiHa), and that *STAT3* knockdown was associated with decreased cell migration and proliferation.

Taken together, these data suggested that SNHG12 plays an important role in promoting tumorigenesis and metastasis in cervical cancer via sponging of miRNAs, such as miR-125p and miR-424-5b. Moreover, since *STAT3* is required by cells to regulate various biological processes, modulation of the components of the SNHG12/miR-125b/*STAT3* signaling axis may reduce the progression of cervical cancer.

### Nasopharyngeal Carcinoma (NPC)

Liu et al. (35) evaluated the expression of SNHG12 in 129 human NPC tissue samples and found it to be up-regulated by ~1.5-fold compared to adjacent normal tissues using qRT-PCR (Table 1) (35). Up-regulated levels of SNHG12 correlated with clinical stage and grade, with no significant correlation with other clinical or pathological characteristics. Moreover, using Kaplan-Meier curves, the authors showed that high SNHG12 expression was correlated with shorter overall survival times (35). Likewise, the evaluation of SNHG12 expression in five different NPC cell lines revealed its up-regulation in CNE1 cells and SUNE1 cells by ~10-fold and ~7.7-fold, respectively, as compared to the control cell line, NP69 (Table 1). Moreover, immunoblot analysis revealed that SNHG12 over-expression was associated with increased levels of protein expression, such as Notch1 and its downstream target proteins p21 and Hes family BHLH transcription factor 1 (Hes1) in NPC cell lines (Figure 2) (35). Following these observations, the authors found that SNHG12 knockdown by siRNA increased the apoptotic rate, and decreased the proliferative and metastatic potentials of the NPC cell lines (CNE1 and SUNE1), presumably by inhibiting Notch signaling. Thus, a positive correlation was established between the expression of SNHG12 and the resultant activation of the Notch signaling pathway (35). In summary, these results provide evidence that SNHG12 may serve as a biomarker for early detection of NPC and/or as a target for more effective treatment strategies for NPC patients.

### Osteosarcoma

Osteosarcoma is the most common primary malignant bone tumor and a major cause of cancer-associated fatality rates in children and adolescents (64). The annual incidence is ~3.4 cases per million people with a peak incidence occurring in late adolescence (45). However, the 5-year survival rates in localized and metastatic osteosarcoma have stagnated since the 1980s (65), presumably due to the lack of advances in chemotherapeutic treatment strategies that can prevent the chemoresistance that often occurs with the traditional therapies. This highlights the urgent need to formulate new therapeutic strategies, which may be provided by identifying potential molecular targets involved in the development of osteosarcoma.

The expression of SNHG12 was examined by three different research groups in 20, 31, and 32 doxorubicin-resistant (DXR) osteosarcoma tissue samples and it was found to be up-regulated by ~3.4-fold, ~2.9-fold, and ~2.4-fold, respectively, as shown in Table 1 (11, 29, 46). Moreover, SNHG12 abundance was associated with various clinical parameters, including advanced tumor grade, tumor size, lymph node metastasis, and Enneking staging, while its over-expression was negatively associated with overall survival rate. In addition, SNHG12 expression in different osteosarcoma cell lines was found to be up-regulated by ~6.2-fold in 143B cells, up to ~3.9-fold in MG63 cells, ~5.4-fold in MG63/DXR cells, and up to ~5.6-fold in U2OS cells compared to normal human osteoblast cell lines (11, 29) (Table 1).

It is known that SNHG12 can act as a ceRNA for various miRNAs, including miR-125b (33), miR-199a/b-5p (8), and miR-320 (52). Further analysis identified and confirmed miR-320a and miR-195-5p as direct downstream targets of SNHG12 that regulated the expression of the *MCL-1* and *NOTCH2* genes, respectively, via sponging (Figure 2). Notch 2 is an important receptor in Notch signaling and has been shown to regulate metastasis and tumorigenesis of osteosarcoma (11). Immunoblot analysis revealed that over-expression of *Notch 2* in osteosarcoma cell lines up-regulated protein levels of Notch intracellular domain (NICD) and Hes1, which are involved in Notch signaling (Figure 2). SiRNA-mediated knockdown of *MCL-1* promoted apoptosis of osteosarcoma cells by increasing their sensitivity toward the anti-cancer drug, doxorubicin. *MCL-1* is an anti-apoptotic member of the Bcl-2 protein family and a key factor in conferring resistance to some cancer types to conventional chemotherapy (29). Consistent with the above findings, it was shown that SNHG12 depletion resulted in over-expression of miRNA-320a and reduced the invasiveness and proliferative capacity of the osteosarcoma cell lines, 143B, U2OS, MG-63, and MG-63/DXR (11, 29, 46). In addition, depletion of SNHG12 increased the susceptibility of U2OS and MG-63 osteosarcoma cells to apoptosis and doxorubicin sensitivity, presumably by down-regulating *Notch 2* and *MCL-1* (11, 29). Moreover, immunoblot analysis showed that the levels of cell cycle progression-related proteins (CDK4, CDK6, and CCND1) were increased (4) in si-SNHG12 transfected U2OS and 143B cell lines as shown in Figure 2.

In a study by Ruan et al. (46), it was reported that SNHG12 acted as an oncogene by regulating the expression of the *Angiomotin (AMOT)* gene (Figure 2). The *AMOT* gene directly regulates Rac1*-*GTPase, and enhances cell migration and stabilizes new blood vessels by activating the ERK pathway (66, 67). Consistent with these reports, combined results of qRT-PCR and immunoblots revealed that the over-expression of SNHG12 correlated with increased *AMOT* gene expression, which further promoted the proliferation and migration capacity of osteosarcoma cells.

These findings suggest that SNHG12 is involved in the etiology of osteosarcoma via the miR-320a/MCL-1 axis, the miR-195-5p/Notch2 axis, and *AMOT* gene up-regulation, and thus might serve as a potential biomarker/target for the diagnosis and/or treatment of osteosarcoma.

#### Roles of SNHG12 in Other Cancers

Apart from the cancer types discussed above, very recently SNHG12 has also been implicated in several other cancers (Table 1); for example, prostate cancer, ovarian cancer, renal cell carcinoma, and lymphoma, discussed below.

Two independent studies have shown that SNHG12 levels were up-regulated (~1.8-fold) in paired human prostate cancer (PCa) tissue samples compared to normal adjacent tissue, and (~2.3-fold) in the DU145 prostate cancer cell line compared to normal prostate cells (47, 48). This upregulation was found to be positively correlated with positive lymph nodes, Gleason score, and advanced residual tumor grade along with poorer recurrence-free survival (47, 48). Moreover, high SNHG12 levels were also correlated with low levels of miR-133b (47) and miR-195 (48), which are known to be involved in regulating proliferation, migration, invasion, and the cell cycle of PCa by enhancing the p53 (47) and Wnt/β-catenin signaling pathways (48). Thus, suggesting that SNHG12 may have potential as a prognostic as well as a diagnostic biomarker for PCa.

Very recently it was demonstrated that SNHG12 was aberrantly up-regulated (~1.75-fold) in renal cell carcinoma cells compared to control samples (49). Corresponding to this upregulation, the high expression of SNHG12 was correlated with poor prognosis (49). Furthermore, decreased SNHG12 levels inhibited cell viability and anchorage-independent growth, and induced apoptosis. However, these SNHG12 silencing-repressive effects were partially reversed by increased miR-199a-5p expression. Corresponding to these results, further analysis revealed that SNHG12 promoted renal cell carcinoma by modulating *HIF1*α expression via the sponging of miR-199a-5p (49).

Sun and Fan (50) reported that SNHG12 was up-regulated (~1.7-fold) in ovarian cancer (OC) tissues as compared to the normal adjacent tissues, and a similar expression was observed in OC cell lines as compared to a normal ovarian epithelial cell line (50). Moreover, SNHG12 abundance was found to be associated with increased metastasis, high-grade tumor (stage III-IV), low overall survival rate, and poor prognosis. Bioinformatics prediction identified *SOX4* as a target of miR-129 which in turn served as a target for SNHG12 (50). Similar to previous results, SNHG12 overexpression enhanced the proliferative and migratory capacity of cells by inducing the expression of SOX4 via repressing the expression of its target miRNA (miR-129) (50).

Zhu et al. (51) studied the regulatory mechanism of SNHG12 in conferring multidrug resistance (MDR) to Natural Killer/T-cell lymphoma (NKTCL). It was observed that SNHG12 expression was increased (~1.3-fold) in NKTCL tissues of stages 3/4 as compared to the NKTCL tissues of stages 1/2, and reactive hyperplasia of lymph node (RHLN) was positively associated with clinical grading (stage 3/4) (51). C-Myc was identified as a direct transcriptional regulator of SNHG12 where its overexpression aided in the proliferation of cells, increased MDR-associated protein levels (P-gp), and inhibited sensitivity to cisplatin (CCDP) by binding to the promoter sequence of SNHG12 thereby promoting its expression (51). Since c-Myc induced upregulation of SNHG12, promoted NKTCL, and inhibited its drug sensitivity, SNHG12 may act as a therapeutic target and novel biomarker for CCDP-resistant NKTCL patients.

### Potential Roles of SNHG12 in Drug Resistance

SNHG12 has been reported to play an important role in MDR in tumor cells. For example, Wang et al. (25) performed qRT-PCR analyses in the NSCLC drug-resistant cell lines, A549/DDP, A549/PTX, and PC9/AB2, and found an ~5-fold, ~4.8-fold, and ~5.5-fold higher expression of SNHG12, respectively, and an ~2.5-fold lower expression of miR-181a compared to their parental cell lines A549 and PC9 (25) (Table 1). Moreover, silencing of SNHG12 using siRNA increased the expression of miR-181a, which correlated with improved sensitivity of the resistant cell lines A549/DDP, A549/PTX, and PC9/AB2, to cisplatin, paclitaxel, and gefitinib, respectively. Similar results were observed in a separate study with increased sensitivity of A549/DDP human tumor xenografts in athymic mice to cisplatin treatment (25). Evidence suggested that silencing of SNHG12 suppressed the expression of *MAPK1* and *MAP2K1*, and upregulated miR-181a further contributing to the inhibition of the MAPK/Slug pathway. Thus, it is possible that SNHG12 may also influence the unfolded protein response (UPR), which is stabilized in a large variety of cancers in a MAPK-dependent manner (68, 69). Many tumor cells exploit UPR via MAPK for the polarization of tumor-associated macrophages and T cells and enhanced angiogenesis (70). p38 MAPK is an example of a protein in the MAPK family that is potentially involved in vascular endothelial growth factor (VEGF)-mediated angiogenesis (70) and functional exhaustion of CD4 and CD8+ T cells. Our recent study has suggested that intratumoral inhibition of p38 MAPK influenced M1 re-tuning of tumor-induced macrophages and decreased the levels of VEGF and TGF-beta in neuroendocrine tumors of the pancreas (71). Thus, from this point of view, it is likely that inhibition of SNHG12 may also influence angiogenesis and depolarization of tumor-primed refractory immune cells contributing to immune restoration, though further studies are warranted.

## Conclusions and Future Perspectives

Based on the literature discussed above, it is evident that pharmacological targeting of SNHG12 may serve as a potential alternative strategy for the diagnosis and treatment of a variety of cancers. It is anticipated that inhibiting SNHG12 may also influence the unfolded protein response, which is at the interface with MAPK signaling, contributing to stress, and angiogenic responses. It is also anticipated that targeting SNHG12 may improve cancer-directed interventions by mitigating UPR and MAPK signaling in both tumors as well as non-tumorous components of tumor-micro milieu. In this context, it would be worthwhile to investigate the extent to which inhibiting SNHG12 might also augment spontaneous effector immune responses and attenuate resistance and relapse in cancer patients. Further, SNHG12 profiling may provide a useful component of personalized medicine for future cancer-directed interventions. Thus, we propose SNHG12 as a potential candidate for cancer-directed interventions and that targeting SNHG12 may allow advances in the diagnoses, prognosis, and/or treatment of a variety of cancer types.

## Author Contributions

ST, VA, DR, RS, AA, and AJ conceived the idea. ST, VA, KV, HP, and AJ wrote the majority of the manuscript. ST, VA, DR, US, and AK composed the figures and table. Critical revisions were made by ST, VA, US, AK, HP, KV, and AJ. All authors read and approved the final manuscript.

### Conflict of Interest Statement

The authors declare that the research was conducted in the absence of any commercial or financial relationships that could be construed as a potential conflict of interest.
